# A call for global governance of biobanks

**DOI:** 10.2471/BLT.14.138420

**Published:** 2014-11-24

**Authors:** Haidan Chen, Tikki Pang

**Affiliations:** a College of Humanities and Development Studies, China Agricultural University, Beijing, China.; b Lee Kuan Yew School of Public Policy, National University of Singapore, 469 C Bukit Timah Road, Singapore 259772, Singapore.

## Abstract

The progress in genomic research has led to increased sampling and storage of biological samples in biobanks. Most biobanks are located in high-income countries, but the landscape is rapidly changing as low- and middle-income countries develop their own. When establishing a biobank in any setting, researchers have to consider a series of ethical, legal and social issues beyond those in traditional medical research. In addition, many countries may have inadequate legislative structures and governance frameworks to protect research participants and communities from unfair distribution of risks and benefits. International collaborations are frequently being created to support the establishment and proper running of biobanks in low- and middle-income countries. However, these collaborations cause cross-border issues – such as benefit sharing and data access. It is thus necessary to define and implement a fair, equitable and feasible biobank governance framework to ensure a fair balance of risks and benefits among all stakeholders.

## Résumé

Les progrès de la recherche génomique ont entraîné une augmentation de l'échantillonnage et de la conservation des échantillons biologiques dans les biobanques. La majorité des biobanques sont situées dans les pays à revenu élevé, mais le paysage évolue rapidement puisque les pays à revenus faible et intermédiaire développent leurs propres biobanques. Lors de la création d'une biobanque quel que soit le lieu, les chercheurs doivent prendre en compte un ensemble de questions éthiques, juridiques et sociales qui vont au-delà des questions rencontrées dans la recherche médicale traditionnelle. En outre, de nombreux pays peuvent présenter des structures législatives et des cadres de gouvernance insuffisants pour protéger les personnes participant à la recherche et les communautés de la répartition inéquitable des risques et des bénéfices. Les collaborations internationales sont fréquemment mises en place pour soutenir l’établissement et le fonctionnement approprié des biobanques dans les pays à revenus faible et intermédiaire. Cependant, ces collaborations génèrent des problèmes transfrontaliers, tels que le partage des bénéfices et l'accès aux données. Il est donc nécessaire de définir et de mettre en œuvre une gouvernance des biobanques équitable et réalisable pour assurer un juste équilibre des risques et des bénéfices entre tous les acteurs.

## Resumen

Los avances en la investigación genómica han dado lugar a una mayor toma y almacenamiento de muestras biológicas en biobancos. La mayoría de los biobancos se encuentran en países de ingresos altos, pero el panorama está cambiando rápidamente a medida que los países de ingresos bajos y medios desarrollan sus propios biobancos. A la hora de establecer un biobanco en una ubicación cualquiera, los investigadores deben tener en cuenta una serie de cuestiones éticas, legales y sociales más allá de las cuestiones de la investigación médica tradicional. Además, es posible que muchos países no cuenten con estructuras legislativas adecuadas y dispongan de marcos de gobierno para proteger a los participantes de la investigación y a las comunidades frente a la distribución injusta de riesgos y beneficios. Por ello, con frecuencia se crean colaboraciones internacionales para apoyar el establecimiento y funcionamiento adecuados de los biobancos en países de ingresos bajos y medios. No obstante, estas colaboraciones provocan problemas transfronterizos como el reparto de beneficios y el acceso a los datos. Por tanto, es necesario definir y poner en práctica un marco de gobernanza de los biobancos justo, equitativo y viable para garantizar un equilibrio justo de riesgos y beneficios entre todos los interesados.

## ملخص

أدى التقدم في بحوث الجينوم إلى الزيادة في جمع العينات وتخزين العينات البيولوجية في البنوك البيولوجية. وتقع معظم البنوك البيولوجية في البلدان المرتفعة الدخل، غير أن المشهد يتغير بشكل سريع نظراً لإنشاء البلدان المنخفضة والمتوسطة الدخل بنوكاً بيولوجية خاصة بها. وعند إنشاء بنك بيولوجي في أي منطقة، يتعين على الباحثين النظر في مجموعة من المسائل الأخلاقية والقانونية والاجتماعية التي تتجاوز المسائل التي تتضمنها البحوث الطبية التقليدية. بالإضافة إلى ذلك، قد تكون لدى العديد من البلدان هياكل تشريعية وأطر لتصريف الشؤون غير كافية لحماية المشاركين في البحوث والمجتمعات المحلية من التوزيع غير المنصف للمخاطر والفوائد. ويجرى في الغالب إنشاء أوجه التعاون الدولي لدعم إنشاء البنوك البيولوجية وإدارتها بالطريقة المناسبة في البلدان المنخفضة والبلدان المتوسطة الدخل. ومع ذلك، تتسبب أوجه التعاون هذه في مسائل عابرة للحدود - مثل مشاركة الفوائد وإتاحة البيانات. ومن ثم، من الضروري تحديد إطار منصف وعادل ومجد لتصريف شؤون البنوك البيولوجية بغية ضمان توازن منصف للمخاطر والفوائد فيما بين جميع أصحاب المصلحة.

## 摘要

基因组研究的发展已导致生物样本库生物样品采样和存储的增加。大多数生物样本库位于高收入国家，但是因为中低收入国家也在发展自有的生物样本库，现在情况也在快速变化。在任何条件中建立生物样本库时，研究人员都必须考虑一系列超越传统医学研究的伦理、法律和社会问题。此外，许多国家可能不具有充分的立法结构和治理框架来保护研究参与者和社区免受风险和收益的不公平分布。国际合作被频频发起，来支持中低收入国家生物样本库的建立和良好运营。然而，这些合作导致跨境问题，如利益共享和数据访问。因此，有必要定义和实现一个公平、公正和可行的生物样本库治理框架，以确保所有利益相关者之间风险和收益的公平平衡。

## Резюме

Прогресс в геномных исследованиях привел к увеличению отбора и хранению биологических образцов в биобанках. Большинство биобанков расположено в странах с высоким уровнем доходов, но ситуация быстро меняется, так как страны с низким и средним уровнем доходов разрабатывают свои собственные биобанки. При организации биобанка в любых условиях исследователи должны рассмотреть ряд этических, правовых и социальных вопросов, помимо тех, которые уже учтены в традиционных медицинских исследованиях. Кроме того, многие страны могут иметь несовершенные законодательные и управленческие структуры для защиты участников исследований и сообществ от несправедливого распределения рисков и выгод. Для поддержки становления и надлежащей работы биобанков в странах с низким и средним уровнем доходов часто используются международные схемы сотрудничества. Однако подобное сотрудничество вызывает определенные трансграничные проблемы, такие как совместное использование выгод и доступ к данным. Таким образом, необходимо разработать и внедрить честную, равноправную и осуществимую структуру управления биобанками для обеспечения справедливого сбалансированного распределения рисков и выгод между всеми заинтересованными сторонами.

## Introduction

The introduction of genomic technology has led to a biomedical revolution. Whole-genome sequencing and genome-wide association studies have become powerful tools to investigate environmental, genetic, social and behavioural determinants of human diseases.^1^^,^^2^ Many countries have set up biobanks to collect human biological samples and their associated data for genomic research and public health purposes. To maximize the utilization of biobanking resources, regional and transnational biobank networks, such as the BBMRI-ERIC (Biobanking and Biomolecular Resources Research Infrastructure), the International HapMap Project and the International Cancer Genome Consortium, have been established.^3^^–^^5^ Although genetics and genomics have contributed to better understanding of causes and mechanisms of human diseases, some researchers are concerned that genetic research conducted to date has mainly focused on the health needs of high-income countries, thus increasing health inequity between people in poor and rich nations.^6^^–^^8^ Low- and middle-income countries are benefiting less than high-income countries from the applications of epidemiological and genetic research. It has been suggested that the disadvantage could partly be attributable to the lack of biobanks and large cohort studies in poorer countries.^9^ To find indigenous solutions to health improvement, biobanks have recently been set up in several developing countries (e.g. China, Gambia, India and Mexico).^10^^–^^14^

The establishment and proper running of a biobank can be perceived as an overwhelming task, since researchers have to consider a series of ethical, legal and social issues, such as informed consent, benefit sharing, confidentiality, ownership, commercialization and public participation.^15^^–^^18^ Building transnational biobank networks is even more difficult, as these require sharing of samples and interoperability of data in a mutually-applicable ethical and legal framework. However, such frameworks differ between countries.^19^ Compared with the situation in high-income countries, where the ethical, legal and social issues of biobanks have been debated, researchers in low- and middle-countries are less experienced in coping with these issues.^12^^,^^13^^,^^20^^,^^21^ The fear of exploitation – i.e. unfair distribution of risks and benefits – makes many low- to middle-income countries hesitant about foreign researchers accessing and using their human biological samples and associated data.^22^^–^^25^ Furthermore, research participants may sometimes not be fully aware of the risks of participation.^22^^,^^25^ Therefore, the proliferation of biobanks in low- and middle- income countries has led to ethical, cross-border and benefit-sharing issues not witnessed in other human research areas, due to local culture, religious beliefs and poor awareness of developed countries’ concept of ethics.^26^ These issues may have a negative impact on international research collaborations. In this paper, we argue that it is important to develop a governance framework at the global level to guarantee equity, fairness and justice in biobank collaboration between developing and developed countries.

## Biobanks in developing countries

Biobanks currently exist on every continent, including Antarctica, with most located in North America and Europe.^27^ However, this landscape is changing rapidly.^4^^,^^14^ Some countries, including China, Gambia, Jordan, Mexico and South Africa, have placed great effort into building their own biobanks and biobanking networks.^10^^–^^14^ In Table 1, we present the aim of biobanks with publicly available information and how these biobanks are funded. All the selected biobanks have partnered with facilities in high-income countries. The Kadoorie Study of Chronic Disease in China and the Mexico City Prospective Study collaborate with Oxford’s Clinical Trial Service Unit and Epidemiological Studies Unit.^28^^,^^29^ The KHCCBIO project in Jordan, that will collect cancer specimens within the country and from its neighbouring countries, collaborates with Trinity College Dublin, Biostór Ireland and Accelopment AG, Switzerland.^30^ The centralized Gambian National DNA Bank was created with help from the *Centre d'Etude du Polymorphisme Humain*, an international genetic research centre located in Paris, France.^31^ Human Heredity and Health in Africa (H3Africa) is based on a partnership among the African Society of Human Genetics, the National Institutes of Health in the United States of America and the Wellcome Trust.^20^^,^^32^^,^^33^

**Table 1:** Biobanks in developing countries with publicly available information

Name	Year established	Funding	Aim
Mexico City Prospective Study	1994	Mexican Ministry of Health, the Wellcome Trust	A cohort study of 150 000 adults older than 34 years to assess the association between risk factors and common causes of death
Gambian National DNA Bank	2000	United Kingdom Medical Research Council, *Centre d’Etude du Polymorphisme Humain*	The first national DNA bank in Africa with focuses on genetic analysis of infectious diseases such as malaria, HIV and tuberculosis, in western African populations
China Kadoorie Biobank	2004	Kadoorie Charitable Foundation, the Wellcome Trust	To investigate genetic and non-genetic causes of many common chronic diseases in 500 000 Chinese people aged 30–79 years
H3 (Human, Heredity and Health) Africa	2010	African Society of Human Genetics, United States’ National Institutes of Health, the Wellcome Trust	To increase the genomic knowledge in the African population
KHCCBIO	2011	European Union	The first cancer biobank in Jordan, which aims to collect 10 000 specimens from cancer patients in Jordan and its neighbouring countries

DNA: deoxyribonucleic acid; HIV: human immunodeficiency virus; KHCCBIO: King Hussein Cancer Centre Biobank.

Data sources: Klingström,^10^ Lawlor et al.,^11^ Rudan et al.,^12^ Sgaier et al.^13^ and Vaught et al.^14^

The establishment of biobanks is an important step towards establishing national genomics research programmes. However, development of biobanks can face challenges. Maintaining these biobanks and producing effective scientific outcomes based on the biobanking resources are not easy without a proper framework and the capacity to manage biobanks. In addition, some countries – such as China and South Africa – lack adequate legislative structures and governance frameworks that regulate the use and development of biobanks.^20^^,^^29^

## Cross-border issues

Facilities in high-income countries conducting human genetic research may see an advantage in examining human samples from populations with rich genetic diversity in low- and-middle income countries. The samples can either be shipped from the biobank in the low- or middle-income country to the research facility or researchers can come and collect the samples from the biobank.^21^ These cross-border flows of biological samples and data are troublesome for low- and middle-income countries, since many of these countries have poor or absent medical and patents laws and/or regulatory frameworks. The lack of legislative structures makes both countries and their people vulnerable to exploitation.^25^

In December 2000 *The Washington Post* published a six-part series titled *The body hunters* that surveyed research subjects in China, Africa and Latin America. The research subjects claimed they did not receive the expected benefits – such as health care services – when participating in medical research led by high-income countries.^34^ There have also been reports of researchers from high-income countries collecting blood samples from Hagahai people in Papua New Guinea, Havasupai people in Arizona, United States of America, and the Karitiana people in Brazil without securing proper informed consent. The participants reported that they were disappointed not to receive the benefits they expected and felt they deserved, such as financial compensation and medicines.^24^

In India, although the government issued regulations against biopiracy in 2002, this was poorly implemented and biological samples are still shipped abroad for studies without the proper approval from authorities.^35^ A systematic review of all human genetic studies using Cameroonian deoxyribonucleic acid (DNA) samples published between 1989 and 2009, found that only 14% of Cameroonian institutions and 28% of Cameroonian authors were associated with any of the identified 50 articles. Moreover, very few studies were on the most common genetic diseases in African populations. Almost all of the Cameroonian DNA samples are stored outside Africa.^21^

In genetic research, benefit-sharing issues are usually central when it comes to possible exploitation cases. In general, benefits can be shared at two levels: (i) at an individual level; and (ii) at a community, tribe or national level.^36^ Benefits can also be shared directly and indirectly. Direct benefits include access to medical care for the participating research subjects and/or communities. Indirect benefits include research-capacity building, such as publications, fund-raising, research staff training and development of a stronger scientific culture. Data sharing in genomic research and human biobanks comprises one form of benefit sharing, even though there are issues with data sharing – such as who owns the data, which third parties can benefit and who decides what can be shared. Researchers may also gain financial benefits, personal recognition and reputation through access to and commercialization of biobanking resources, which could potentially violate the interests of research participants.^37^ Unfair benefit-sharing with local participants and communities may constitute exploitation, and contribute to a public distrust of biomedical research. In addition, poor consent procedures and inadequate engagement, both at an individual and community level complicate the relationship between researchers and participants.^23^^,^^24^

Benefit-sharing issues in cross-border flows of samples and data have previously been debated. Several studies have discussed and made suggestions for fair benefit-sharing in genetic research collaboration between countries.^23^^,^^25^^,^^29^^,^^36^^,^^38^^,^^39^ However, the conceptual and practical problems of benefit-sharing remain unsolved. Some international organizations have developed ethical and legal policies to promote benefit sharing and data access – such as the Human Genome Organization Ethics Committee’s *Statement on Benefit Sharing* (2000), the United Nations Educational, Scientific and Cultural Organization’s (UNESCO’s) *International Declaration on Human Genetic Data* (2003) and the Organisation for Economic Co-operation and Development’s *Principles and Guidelines for Access to Research Data from Public Funding* (2007). However, these organizations and policies provide inconsistent and incomplete frameworks, and none of them possess supra-national status, authority or enforceability.^37^

## Global governance of biobanks

Low- and middle-income countries have weaker research capacity and governance mechanisms for biobanks than high-income countries.^12^^,^^40^ It is important to develop a feasible and equitable governance framework at the global level to guarantee benefit sharing in biobank collaboration. The potential commercial benefits resulting from access to the data of biobanks underscores the urgent need for such a framework.

International initiatives – such as the Public Population Project in Genomics and Society,^41^ the International Society for Biological and Environmental Repositories^42^ and the International Agency for Research on Cancer^43^ – have offered governance structures, best practices and guidelines to promote the internationalization and standardization of biobanks. An international research group has created the ELSI 2.0 initiative to accelerate the translation of ethical, legal and social knowledge into policy and practice. ELSI 2.0 invites people working with biobanks, policy-makers, funders, the public and other stakeholders to be engaged in ethical, legal and social research.^44^ In addition, international organizations should harmonize the multiple existing standards, best practices and guidelines, and consolidate these into a single global governance framework for biobank operation and collaboration.

We propose a provisional global governance framework for biobanks that includes the following six key elements: (i) respecting participants and donors of biological samples, and protecting their privacy and confidentiality; (ii) informing participants and donors of potential risks through initial consultations; (iii) sharing samples, data and benefits in a fair, transparent and equitable manner; (iv) ensuring quality and interoperability of samples and their associated data; (v) improving public awareness, trust and participation in biobanks; and (vi) defining the role of the private sector in the use of knowledge derived from biobank operations.

As a step towards global governance, the Global Alliance for Genomics and Health was formed in 2013 to convene global stakeholders from more than 210 leading institutions across different sectors. The alliance aims to enable responsible data-sharing for genomic innovation and discovery. The Global Alliance proposes a provisional *Framework for Responsible Sharing of Genomic and Health-Related Data*,^45^ which includes all of the six elements we propose. The framework is currently available from http://genomicsandhealth.org/ and is open for comments, and provides a setting for further discussions among key stakeholders and interested parties. A key question will be the legitimacy and implementation of any proposed framework or guidelines on data-sharing. We call upon the following organizations to jointly develop a comprehensive global framework to ensure that the benefits of biobanks will be shared by all: the World Health Organization, UNESCO, the World Intellectual Property Organization, the World Trade Organization and the World Medical Association.
